# Supplementary material for: Genome-Scale Identification of Wild Soybean Serine/Arginine-Rich Protein Family Genes and Their Responses to Abiotic Stresses
Source: Int J Mol Sci. 2024 Oct 17;25(20):11175. doi: 10.3390/ijms252011175 (PMC11508973; doi:10.3390/ijms252011175)

Figure S1. Protein–protein interaction networks among GsSR family members. The network nodes represent GsSR proteins, and edges represent protein–protein associations.

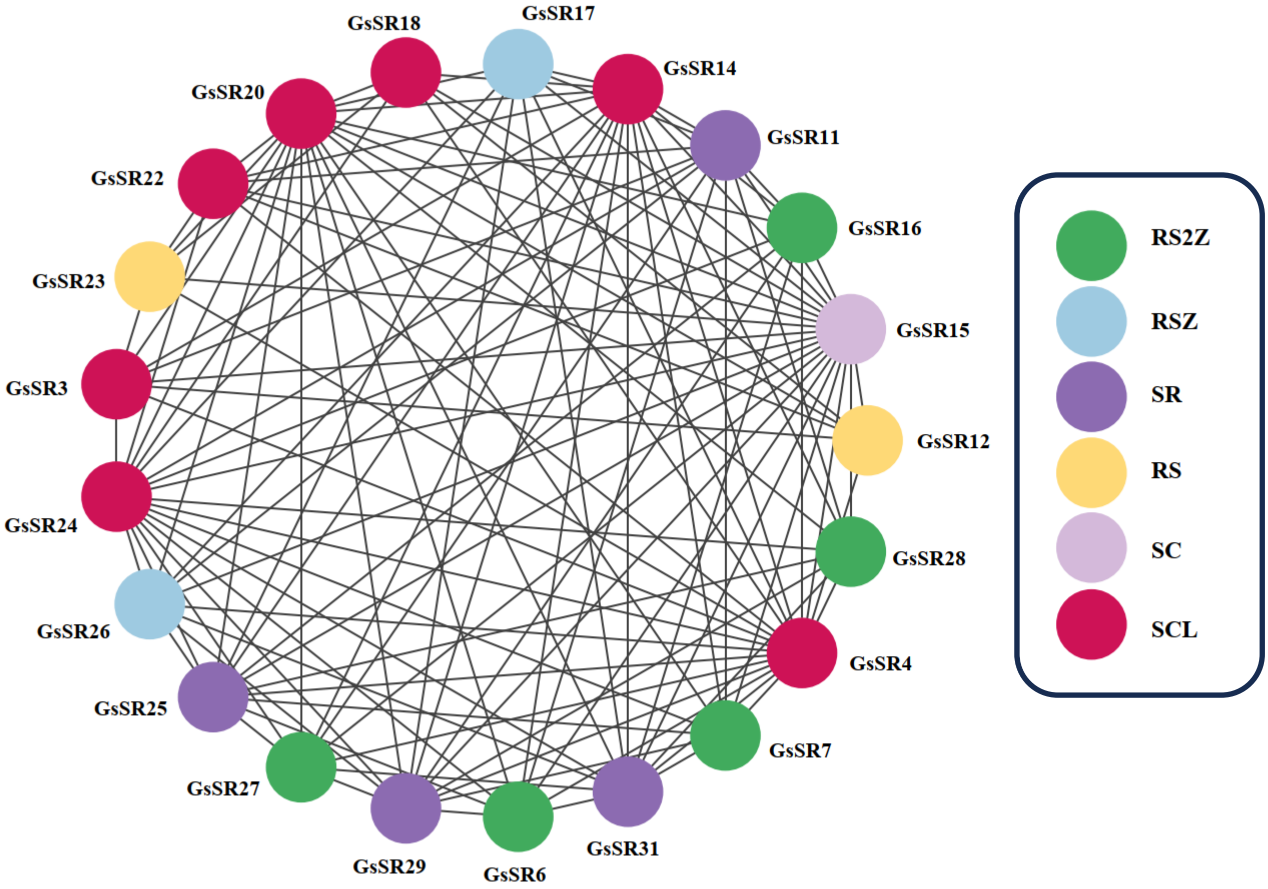

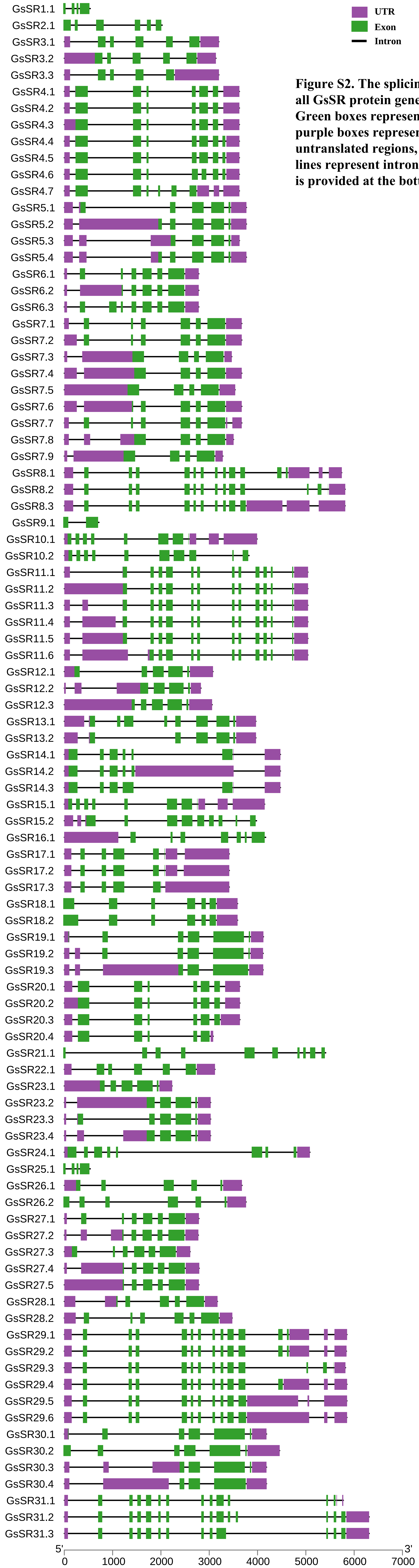

Figure S2. The splicing patterns of all GsSR protein gene transcripts. Green boxes represent exons, purple boxes represent untranslated regions, and black lines represent introns. A scale bar is provided at the bottom.

Figure S3. The statistics of transcript numbers for GsSRs. (A) The distribution of GsSR protein gene numbers based on their transcript numbers; (B) The distribution of transcript numbers of different SR protein sub-families. Table S1. Gene specific primer pairs of GsSRs for Y2H analysis.

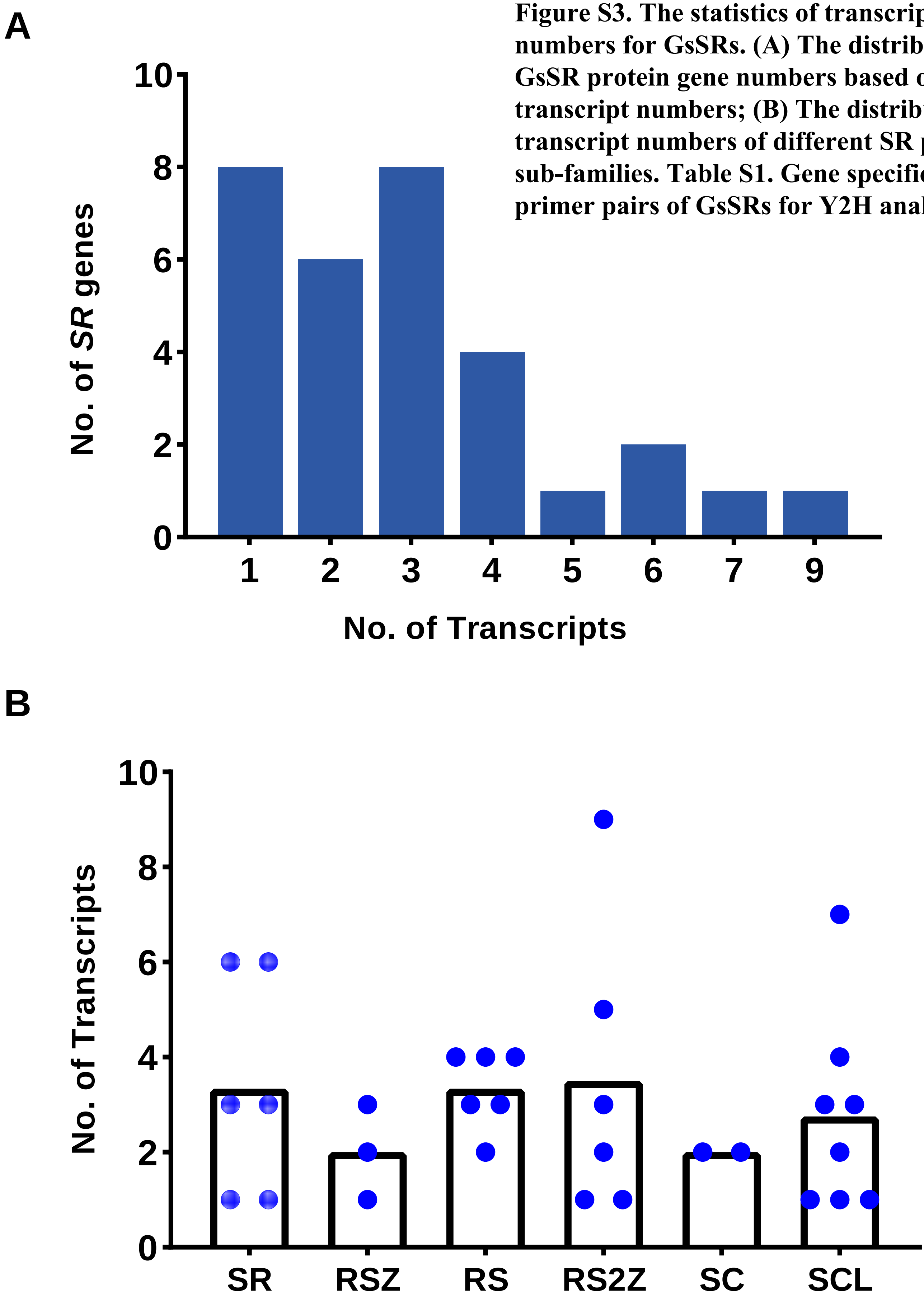

Supplement: Supplementary file 1 [file ijms-25-11175-s001.zip › Supplementary Figures.pdf]
